# Infectious Extensor Tenosynovitis of the Hallucis Longus Tendon: A Case Report

**DOI:** 10.7759/cureus.60384

**Published:** 2024-05-15

**Authors:** Jesse W St Clair, Kara Bragg, Jessica L Mattingly, Jeremy Collado, Brittany C Beel, Ali A Siddiqui, Courtney L James

**Affiliations:** 1 Emergency Medicine, Mayo Clinic, Jacksonville, USA; 2 Orthopedic Surgery, Mayo Clinic, Jacksonville, USA

**Keywords:** case report, pressure ulcer, osteomyelitis, diabetic foot infections, infectious tenosynovitis, hallucis longus, extensor tenosynovitis

## Abstract

Infectious tenosynovitis can involve both flexor and extensor tendons of the extremities. If left untreated, it can lead to high morbidity and mortality. Most emergency providers recognize the signs and symptoms of flexor and extensor tenosynovitis of the hand. However, extensor tenosynovitis of the hallucis longus tendon is a rare condition with a risk of complications similar to infectious tenosynovitis of the hand. This case report describes a presentation of extensor tenosynovitis of the hallucis longus tendon. Clinical suspicion is essential to help the provider not miss this rare condition, which can lead to significant morbidity if not treated promptly or appropriately.

## Introduction

Tenosynovitis, an inflammatory condition affecting a tendon and its sheath, can be caused by trauma, overuse, autoimmune inflammatory processes, foreign bodies, or infection [1]. Treatment options for infectious tenosynovitis include antibiotics and surgical debridement [2]. Infectious tenosynovitis often results from trauma with direct inoculation of bacteria spreading from local tissues or blood. Flexor tenosynovitis is most common and recognized by its characteristic swelling of the affected digit, Kanavel signs, which demonstrate tenderness along the flexor sheath, and pain with passive extension [3]. Extensor tenosynovitis is a less common diagnosis usually seen in the wrist or ankle [4].

In the diabetic foot, septic tenosynovitis is rare. It is most often associated with pressure ulcers in areas of bony prominence and affects the peroneal and Achilles tendons in the heel and the flexor tendons in the foot [5]. Extensor tenosynovitis often lacks typical findings seen in flexor tenosynovitis, which can result in delayed or missed diagnosis [4]. Clinical suspicion is paramount to obtaining appropriate imaging such as magnetic resonance imaging (MRI) and emergent orthopedic consultation for management. Contrast computerized tomography (CT) or ultrasound (US) have lower sensitivity but can be used where MRI is not available [3,6]. Prompt treatment with broad-spectrum antibiotics and orthopedic consultation is essential to prevent complications such as decreased function, tendon sheath rupture, osteomyelitis, necrotizing fasciitis, and amputation [7].

## Case presentation

**Figure 1:**
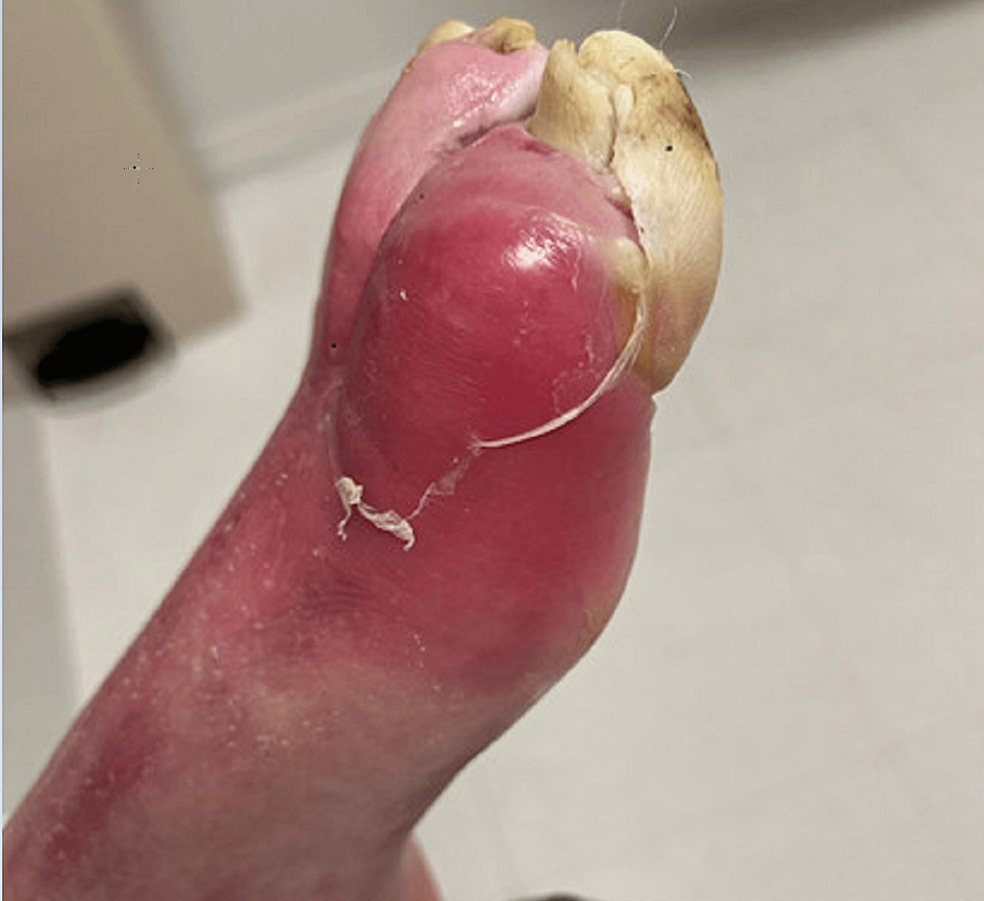
Lateral view of the foot showing erythema and fusiform swelling and purulent drainage of the great toe

**Figure 2:**
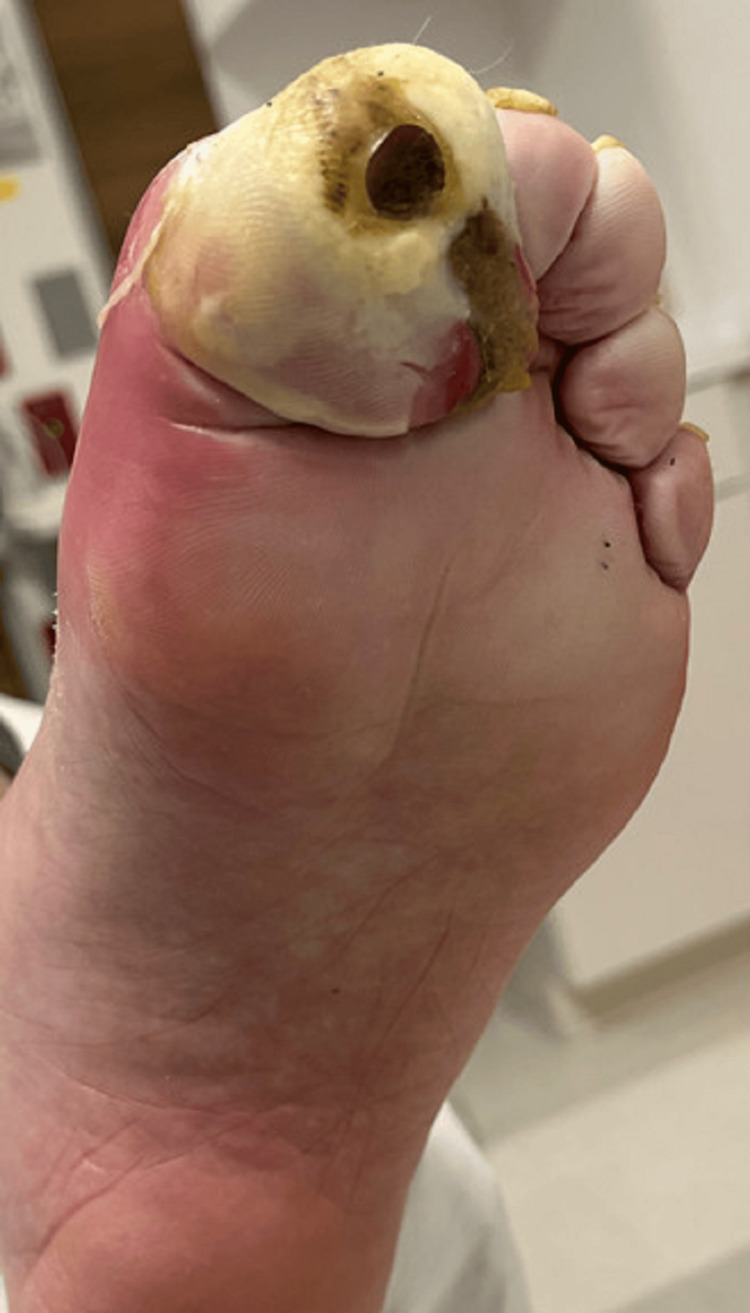
Plantar view of the foot showing ulceration on the great toe

A 57-year-old man with a medical history of type 2 diabetes and hypertension presented to the emergency department with a left great toe wound and pain. Initially, he injured his toe in the shower and subsequently developed erythema, edema, and pain over the course of six days. Twenty-four hours prior to presentation, his pain intensified, and the erythema began to extend to his proximal foot and distal tibia. On physical examination, his left great toe was edematous and erythematous with purulent drainage from the distal aspect of the wound. The dorsal pedis pulse was present. The patient had diminished sensation from neuropathy. There were preliminary stages of ulceration on the plantar side of the great toe that the patient reported to be acute (Figures 1, 2). 

**Figure 3:**
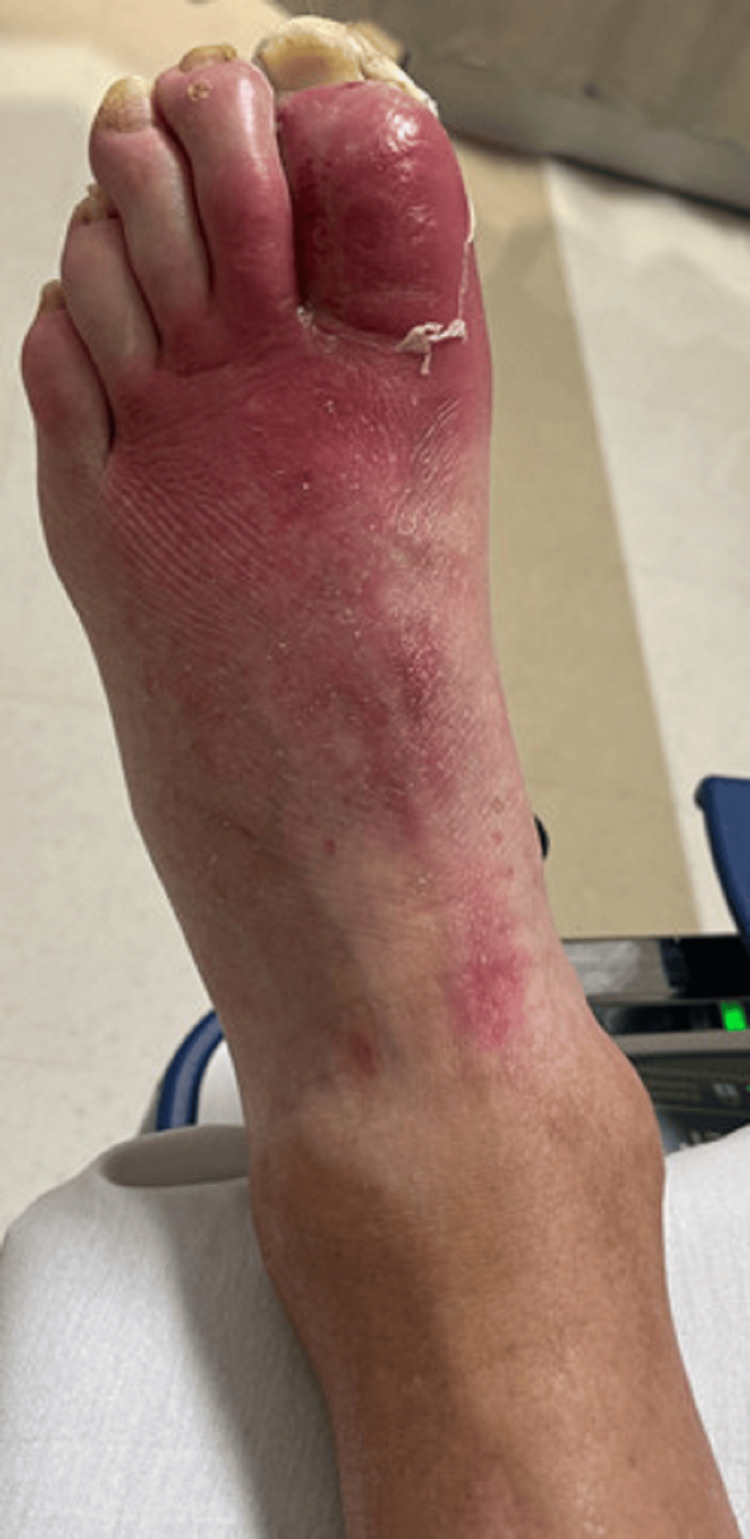
Dorsal view of the foot showing erythema extending in a linear-like fashion to the base of the tibia

There was erythema streaking upward from the dorsal aspect of his foot to the mid-tibial shaft (Figure 3).

The patient had extreme pain in his left great toe on active and passive range of motion. Laboratory studies were remarkable for elevated glucose, hemoglobin A1c, inflammatory markers, leukocytes, and neutrophils, with normal lactate and mild hyponatremia, indicating infection and poorly controlled diabetes (Table 1).

**Table 1:** Laboratory Values in the Emergency Department

Parameter	Patient Result	Reference Range
Sodium (plasma)	131 mmol/L	135–145 mmol/L
Glucose	200 mg/dL	70–140 mg/dL
Hemoglobin A1c	7.8%	4.0–5.6%
Sedimentation rate B	50 mm/hour	0–22 mm/hour
C-reactive protein	170.7 mg/L	<5.0 mg/L
Lactate	1.5 mmol/L	0.5–2.2 mmol/L
Leukocytes	12.9 × 10^9^/L	3.4–9.6 × 10^9^/L
Segmented neutrophils	81%	50.0–75.0%
Absolute neutrophils	10.70 × 10^9^/L	1.56–6.45 × 10^9^/L

**Figure 4:**
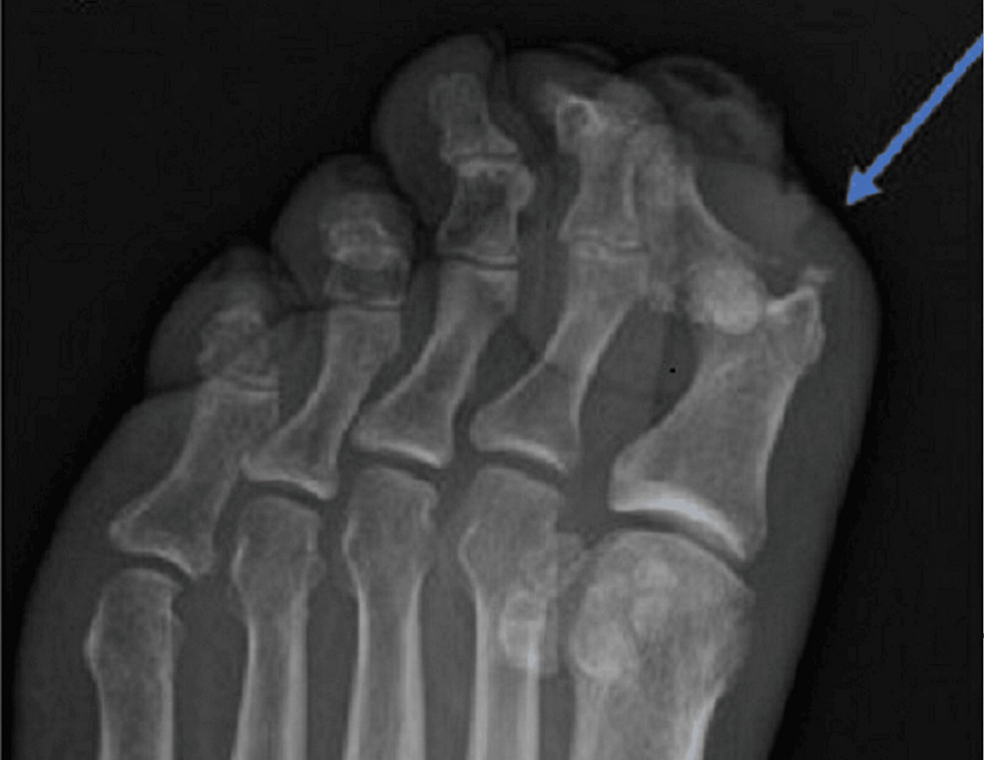
Radiograph showing subluxation of great toe, fracture fragments, and foci of gas Soft tissue swelling around the great toe with irregular foci of soft tissue gas along the distal aspect of the toe, consistent with a wound. No radiopaque foreign body is present. The arrow shows an area of plantar subluxation of the distal phalanx with respect to the proximal phalanx, with several small fracture fragments adjacent to the distal phalangeal base.

Blood cultures were obtained. Radiography of the foot and toe showed soft tissue swelling around the great toe, no radiopaque foreign body, and an irregular focus of soft tissue gas along the distal aspect of the toe. Also noted was a plantar subluxation of the distal phalanx with respect to the proximal phalanx, with multiple acute small fracture fragments adjacent to the distal phalangeal base, but no osseous erosions to suggest osteomyelitis (Figure 4).

**Figure 5:**
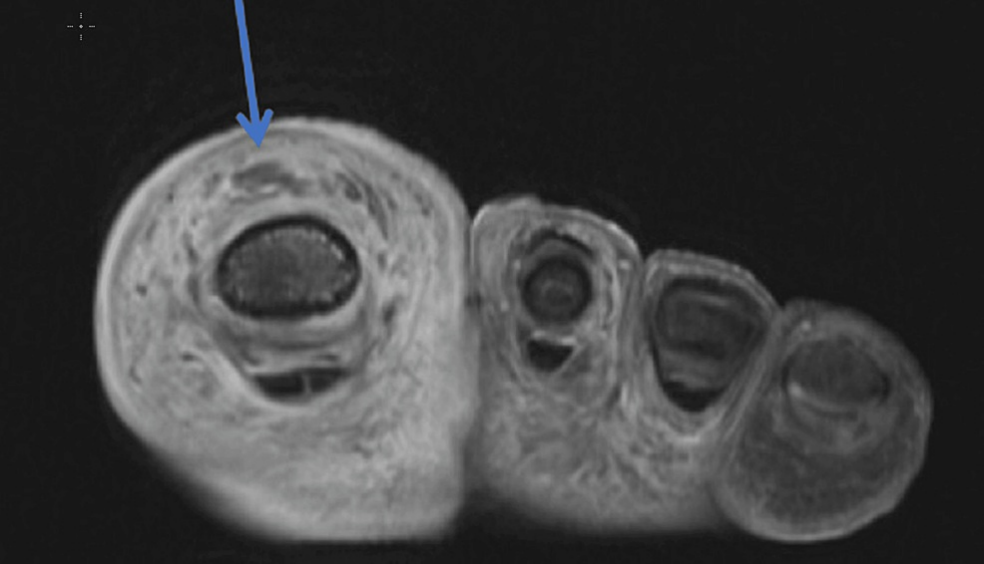
Axial view of the foot showing enhancement and thickening and irregularity of the extensor tendon The arrow shows abnormal synovial thickening and enhancement along the extensor tendon, with an irregular appearance of the tendon.

**Figure 6:**
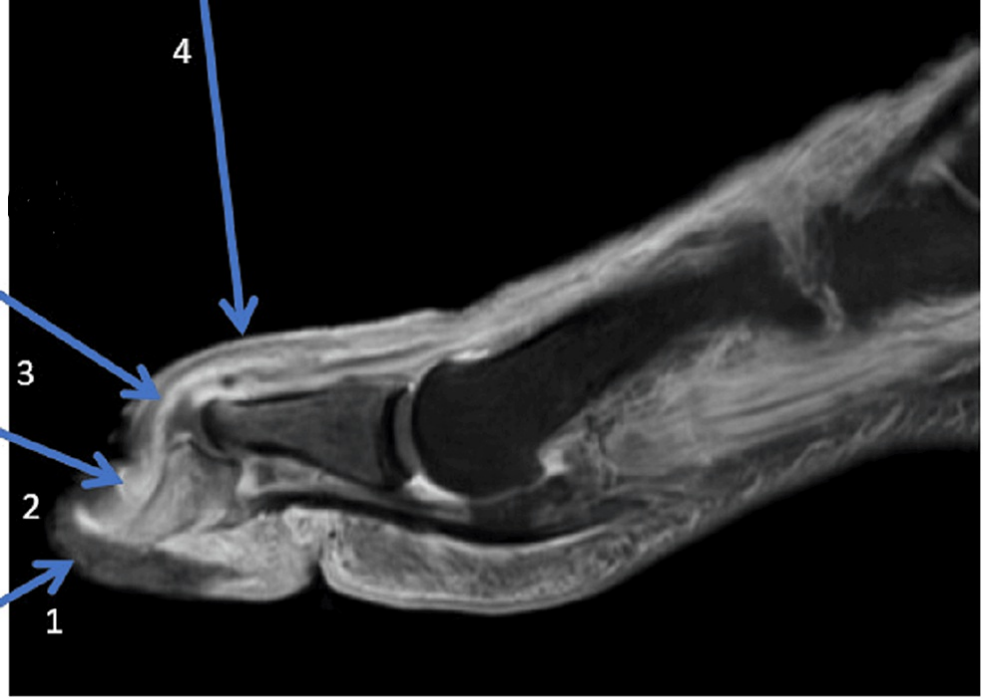
Sagittal view of the foot on MRI demonstrating osteomyelitis, abnormal synovial thickening consistent with tenosynovitis, and tissue loss consistent with plantar ulcer. The image was obtained after reduction of the proximal interphalangeal joint. Arrow 1: Devitalized non-enhancing ulcer along the plantar aspect of the great toe in direct continuity with the tip of the distal phalanx; Arrow 2: Abnormal enhancement in the distal phalanx bone marrow consistent with osteomyelitis; Arrow 3: Thickened joint capsule with fluid in the interphalangeal joint consistent with septic arthritis. Arrow 4: Abnormal synovial thickening and enhancement along the extensor tendon of the great toe.

Given the rapid progression of symptoms in the last 24 hours, the differential diagnosis included sepsis, cellulitis, abscess, osteomyelitis, necrotizing fasciitis, and gangrene. The patient was given vancomycin and cefepime intravenously and admitted to the hospital. Orthopedic surgery and infectious disease were consulted. Recommendations included the option for surgical amputation versus a trial of antibiotic treatment and wound care with debridement. The patient declined amputation in favor of a trial of antibiotic therapy. The great toe was reduced in the emergency department bedside by the orthopedic surgeon, and a post-op shoe was placed. Magnetic resonance imaging was done within 12 hours of admission and showed distal great toe osteomyelitis with interphalangeal joint effusion and enhancement of the synovium along the extensor hallucis longus tendon consistent with septic extensor tenosynovitis (Figures 5, 6).

**Figure 7:**
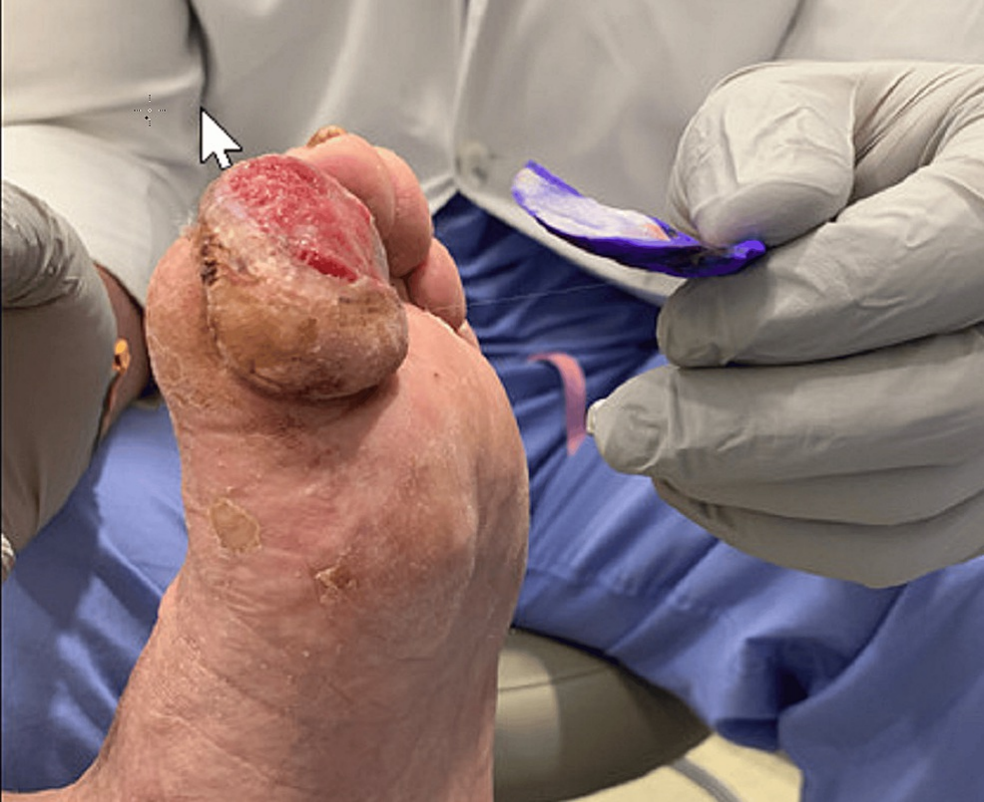
The foot on follow-up visit with orthopedics five weeks after the Emergency Department visit. The toe shows granulation of tissue after debridement.

The patient was treated with intravenous vancomycin and piperacillin-tazobactam and received daily wound care while admitted to the hospital. His blood cultures grew Streptococcus agalactiae. He was treated for 14 days as an inpatient for both osteomyelitis and extensor tenosynovitis with improvement in the infection. He was subsequently discharged on IV vancomycin and daptomycin antibiotic therapy based on blood cultures and sensitivity. These were administered through a peripherally inserted central line catheter for an additional six weeks for concurrent tenosynovitis and osteomyelitis. The patient also had wound care debridement daily during his hospital stay and after his discharge. At his follow-up appointment five weeks later, his infection demonstrated continued improvement, and he maintained full function of his foot and great toe (Figure 7).

## Discussion

Diabetic foot infections can present a diagnostic challenge despite the use of modern imaging and diagnostic tools [8]. Diabetic patients are diagnosed with soft tissue infections or cellulitis early in the disease process, but their foot infections often progress, especially when involving the calcaneus, first, and fifth metatarsal [8]. Timely diagnosis and treatment are essential to prevent complications such as sepsis, osteomyelitis, and rarely septic tenosynovitis [8].

Septic tenosynovitis is an infection that involves the tendon and its synovial sheath, which provides an ideal environment for pathogens to grow in isolation [9]. Typically, these infections involve the flexor tendons of the hand as opposed to the lower extremities [1]. Foreign bodies secondary to trauma are the most common cause of tenosynovitis in the foot [10]. There has been a case reported of a sesamoid bone as the source of tenosynovitis of the flexor hallucis longus tendon [11]. There is no literature to show that fracture fragments could act as a similar irritation mechanism as sesamoid bones. Mechanical tenosynovitis of the extensor tendons of the foot is typically associated with chronic exposure to repeated trauma, such as in marathon runners [12]. Tendonitis in the diabetic foot is most often associated with flexor tendonitis and in association with a prolonged pressure ulcer [5]. Previous case reports also show a correlation with cellulitis, diabetes, and osteomyelitis with peroneal and Achilles tenosynovitis [5].

Trauma in the diabetic foot can lead to cellulitis and osteomyelitis, and this was in the differential for this case study. The emergency clinician should expand their differential to consider septic extensor tenosynovitis even in the absence of traditional triggers for this condition. The patient in this case study had poorly controlled diabetes but lacked risk factors for extensor tenosynovitis such as a foreign body or an overuse injury. The patient did have an early plantar pressure ulcer, but these are often associated with flexor tenosynovitis, not extensor tenosynovitis. Although rare, septic extensor tenosynovitis in a patient with diabetes should be considered and ruled out early to preserve the function of the limb and prevent progression to amputation or tibialis anterior rupture [13].

## Conclusions

Tenosynovitis is a limb-threatening infection of the tendons and synovial sheaths. Classically, it is associated with the flexor tendon sheaths of the hand. However, this case presentation demonstrates that it is possible to have septic extensor tenosynovitis of the foot involving the hallucis longus tendon. It is important for emergency clinicians to have a high index of suspicion when evaluating wounds or infections of the feet, especially in patients with poor peripheral perfusion such as those with diabetes or vasculopathy. Early suspicion will lead to appropriate testing, diagnosis, and specialist involvement. Treatment with broad-spectrum systemic antibiotics and orthopedic consultation is the mainstay of treatment to prevent complications such as decreased function, tendon sheath rupture, osteomyelitis, necrotizing fasciitis, and amputation.
